# Learning health systems on the front lines to strengthen care against future pandemics and climate change: a rapid review

**DOI:** 10.1186/s12913-024-11295-3

**Published:** 2024-07-22

**Authors:** Samantha Spanos, Genevieve Dammery, Lisa Pagano, Louise A. Ellis, Georgia Fisher, Carolynn L. Smith, Darran Foo, Jeffrey Braithwaite

**Affiliations:** 1 Centre for Healthcare Resilience and Implementation Science, Australian Institute of Health Innovation, Macquarie University, 75 Talavera Rd, Sydney, North Ryde, NSW 2109 Australia; 2 NHMRC Partnership Centre for Health System Sustainability, Macquarie University, Sydney, Australia; 3 Faculty of Medicine, Health and Human Sciences, MQ Health General Practice, Macquarie University, Sydney, Australia

**Keywords:** Learning health systems, Learning care systems, Primary care, Emergency department, Implementation science, Review

## Abstract

**Background:**

An essential component of future-proofing health systems against future pandemics and climate change is strengthening the front lines of care: principally, emergency departments and primary care settings. To achieve this, these settings can adopt learning health system (LHS) principles, integrating data, evidence, and experience to continuously improve care delivery. This rapid review aimed to understand the ways in which LHS principles have been applied to primary care and emergency departments, the extent to which LHS approaches have been adopted in these key settings, and the factors that affect their adoption.

**Methods:**

Three academic databases (Embase, Scopus, and PubMed) were searched for full text articles reporting on LHSs in primary care and/or emergency departments published in the last five years. Articles were included if they had a primary focus on LHSs in primary care settings (general practice, allied health, multidisciplinary primary care, and community-based care) and/or emergency care settings. Data from included articles were catalogued and synthesised according to the modified Institute of Medicine’s five-component framework for LHSs (science and informatics, patient-clinician partnerships, incentives, continuous learning culture, and structure and governance).

**Results:**

Thirty-seven articles were included, 32 of which reported LHSs in primary care settings and seven of which reported LHSs in emergency departments. Science and informatics was the most commonly reported LHS component, followed closely by continuous learning culture and structure and governance. Most articles (*n* = 30) reported on LHSs that had been adopted, and many of the included articles (*n* = 17) were descriptive reports of LHS approaches.

**Conclusions:**

Developing LHSs at the front lines of care is essential for future-proofing against current and new threats to health system sustainability, such as pandemic- and climate change-induced events. Limited research has examined the application of LHS concepts to emergency care settings. Implementation science should be utilised to better understand the factors influencing adoption of LHS approaches on the front lines of care, so that all five LHS components can be progressed in these settings.

**Supplementary Information:**

The online version contains supplementary material available at 10.1186/s12913-024-11295-3.

## Introduction

For the last three decades, the performance of modern healthcare systems has remained stagnant. Challenges posed by rising healthcare costs, aging populations, and chronic disease burden have not been sustainably addressed, instead being met with top-down change efforts and fragmented healthcare delivery models that constrain system efficiency and progress [1–3]. Approximately 60% of care is delivered in line with guidelines, 30% of care is of little value, and 10% of care is harmful to patients [3]. Learning health systems (LHSs) have been recommended as a solution to improve the quality of care delivery, by leveraging big data and exploiting knowledge to create continuous improvement [3, 4].

Since the concept of an LHS was discussed in a seminal 2007 publication by the Institute of Medicine (IoM; now the National Academy of Medicine) [5], research interest in LHS development and application has proliferated [6, 7]. In 2013, the IoM defined four key, inter-related components of an LHS: *science and informatics* (access, capture, and synthesis of real-time clinical data and care experiences) *patient-clinician partnerships* (patients, families, and caregivers fully engaged as partners in care)*, incentives* (aligned with continuous improvement and full transparency), and *continuous learning culture* (leadership-driven collaboration and skill-building) [8, 9]. Work in 2020 by Zurynski et al. added a fifth component: *structure and governance,* which included policies, regulations, and governance structures aligned with, and in support of, continuous learning and collaboration [9].

To be sustainable, health systems must be accomplished at using data, embedding knowledge into practice, and improving decision making for healthcare professionals and patients at the front lines of care: primary care and emergency departments. Primary care (PC) is typically the first point of contact for people in healthcare systems across the world, and provides both preventative and curative care [10], while emergency departments (EDs) deal with critical, acute medical incidents that require immediate attention [11].

The pressures on these settings are continually in flux; for example, the COVID-19 pandemic brought an increased volume and complexity of patient presentations, staff shortages, and funding limitations, particularly affecting countries with more fragile health systems [12, 13]. Even prior to the pandemic, PC and ED settings in many countries were under considerable pressure. ED visits have been steadily increasing over the last decade in many health systems, a significant proportion of which report ‘inappropriate’ or non-urgent cases, and which could have been managed in PC [11, 14]. Determinants for the rising demand in emergency care include a rapidly ageing population, low availability of PC providers, and financial constraints on communities in economic crises [11].

Despite arguments to deflect cases from EDs to PC settings, they are under substantial stress. There have been global calls to strengthen PC systems to cope with surges in demand associated with the growing burdens of chronic disease [15]. PC plays a critical role in improving health outcomes, health system efficiency and health equity, and there are persuasive arguments that PC is integral to contributing to economic stability and growth [16, 17]. The urgent need for solutions that improve the quality and safety of care in PC and ED settings is heightened when considering the increasing threats exemplified by COVID-19, and of climate change on health system sustainability [18]. Greater frequency and intensity of extreme weather events, such as bushfires and floods, and the harm and diseases associated with them, place additional pressures on health and social care systems, giving rise to new diseases and exacerbating existing illnesses [19, 20]. Care delivery models must adapt and respond to not only acute events, but to the projected increase in volume and complexity of patients, to build resilience against future pandemics and climate change-induced disasters [19, 21].

Future-proofing the front lines of care against current and new threats relies on developing and implementing systems that can improve and learn on-the-go [22]. LHSs can better adapt to constant change, enabling greater support for high quality care rather than just finding acute and reactive solutions to problems [23]. Although LHS concepts have been much discussed in the literature [4], there are few rigorous formative and summative assessments of them [7]. Understanding how far we have progressed in applying LHS approaches to the front lines of care will aid in formulating LHSs 2.0 – LHSs that are prepared for looming health system threats [21].

### Objective

This rapid review aimed to understand the breadth and range of LHS approaches used in PC and ED settings, the extent to which LHS approaches have been adopted in these key settings, and the barriers and facilitators associated with their adoption.

## Methods

### Review protocol and registration

The rapid review was conducted in accordance with the Cochrane Library guidelines for rapid reviews [24] and followed a registered protocol on PROSPERO (CRD42023416536).

### Search strategy

Comprehensive search strategies were developed to capture LHS concepts as applied to PC and ED settings. Three databases (Embase, Scopus, and PubMed) were searched on 14th March 2023 (see Supplementary Material 1 for Embase search strings). Searches were limited to publications written in English and published from 1st January 2018 to 14th March 2023, to focus on contemporary LHS research from the last five years.

#### Article selection

References were downloaded from databases into Endnote where duplicates were identified and removed. Titles and abstracts were screened within Endnote by two team members (GD, SS) according to inclusion and exclusion criteria (Table 1). PC settings were classified as the first service sought by a patient outside of a hospital or specialist service, including diagnostic and treatment services and long-term care, health promotion, and prevention services [25]. In accordance with Cochrane Library rapid review guidelines [24], 20% of references were independently screened by two reviewers to establish reliability of screening decisions, with the interrater reliability assessed to be sufficiently high (κ ≥ 0.80).

**Table 1:** Inclusion and exclusion criteria for study selection

**Inclusion criteria:**
• Articles published as full-text articles in peer-reviewed journals
• Articles reporting in the context of primary care, including general practice, community services or clinics, and allied health services, or emergency departments
• Articles that had a key focus on one or more components of learning health systems
**Exclusion criteria:**
• Grey literature, unpublished works (theses, preprints), conference abstracts
• Articles not published in English
• Articles published prior to 2018
• Articles that briefly reference primary care or emergency department contexts and that have a more general healthcare systems perspective
• Articles that briefly reference learning health systems, or identify as learning health systems, without providing specific information or commentary on at least one of five components of learning health systems

After title and abstract screening, the full texts of articles deemed potentially relevant were reviewed by three team members (GD, SS, LP), who initially independently screened 20% of articles, assessing for eligibility based on the inclusion and exclusion criteria (Table 1). Each team member then independently screened a third of the remaining 80% of articles, according to Cochrane guidelines [24]. For title, abstract, and full text screening, disagreements were resolved through discussion, and consultation with the broader team (GF, LAE, CLS, JB) as needed.

### Data extraction and synthesis

Data were extracted, organised and synthesised into key categories using a purpose-designed Excel data extraction sheet which was developed a priori by three team members (SS, GD, LP), piloted with a small subset (10%) of articles, and iteratively refined until the final version was reached [25]. Following the pilot of the data extraction sheet, the same three team members extracted data from the remaining 90% of articles, meeting regularly to ensure alignment in information extracted, discuss discrepancies, and reach consensus on data categorisation.

Categories of data extraction included the five components of the Zurynski et al. [9] LHS framework (*science and informatics, patient-clinician partnerships, incentives, continuous learning culture,* and *structure and governance*), article author, publication year, country of publication, study type, setting, LHS definitions (according to the Zurynski et al. [9] definition categories), barriers and facilitators associated with the adoption of LHSs (where applicable), and whether frameworks and/or models were used to guide exploration of barriers and facilitators. Extracted findings were organised and synthesised to identify patterns and explore relationships in the data [26]. Data were also quantified to analyse the extent and distribution of articles across the data extraction categories, and these numerical analyses were tabulated and summarised.

### Quality assessment

As the included articles were broad in their publication design, three appraisal tools were used: 1) Mixed-Methods Appraisal Tool (MMAT) [27]; 2) Scale for the quality Assessment of Narrative Reviews (SANRA) [28]; and 3) Joanna Briggs Institute (JBI) Text and Opinion [29]. Each article was assessed by one of three team members (SS, GD, LP), and where there were any uncertainties, a second team member was consulted to discuss and reach consensus on quality ratings. If an article could not be comprehensively assessed using any of these tools (e.g., field reports describing methodologies), the authorship team met to make a judgement on its quality. This process involved the team discussing potential bias in the design, conduct, and analysis of studies, and whether each criterion was adequately addressed by the authors of the article in question.

## Results

### Search results

**Fig. 1:**
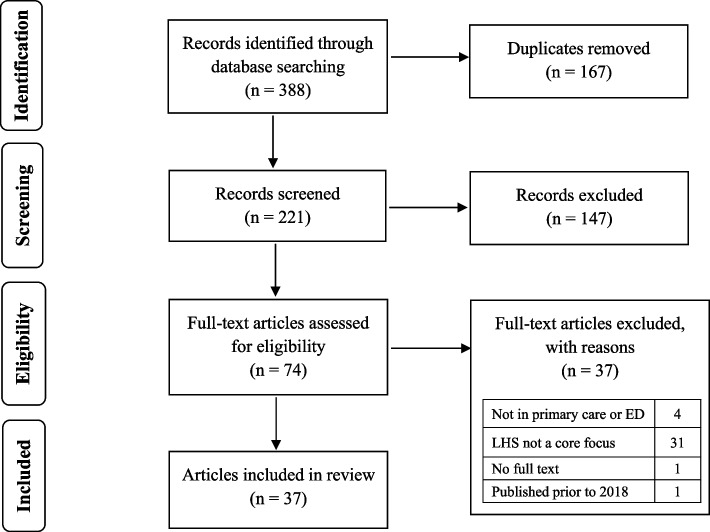
PRISMA flowchart displaying the process of identification and selection of included articles

Figure 1 presents the Preferred Reporting Items for Systematic Reviews and Meta-Analyses (PRISMA) diagram. Database searches yielded 388 references. After removing duplicates, 221 articles were screened by title and abstract against inclusion criteria. Of these, 147 did not meet eligibility criteria, leaving 74 for full-text review. Full text review yielded 37 articles eligible for inclusion (see reasons for exclusion in Fig. 1).

### Characteristics of included articles

This rapid review included 37 articles that examined LHS components within PC (*n* = 32, 86%) and/or ED (*n* = 7, 19%) settings. Table 2 and Supplementary Material 2 display the characteristics of included articles. Most articles were published in North America (*n* = 25; 68%), followed by Europe (*n* = 7; 19%), and Africa (*n* = 3; 8%). The remaining two articles were published in Australia and Asia. Twenty articles (54%) reported on empirical findings from implementation work that progressed health systems toward an LHS [30, 31]. Seventeen articles (46%) were non-empirical and were largely focused on describing the process by which LHSs were adopted [32, 33], discussing the implications and reach of previous work [34, 35], or providing recommendations for future LHSs [36, 37]. See Supplementary Material 3 for the LHS components captured in each article.

**Table 2:** Frequency of article characteristics

Study characteristics (***N*** = 37)	*n* (%)
***Publication year***	
2018–2020	17 (46)
2021–2023	20 (54)
***Study location***	
North America	25 (68)
Europe/UK	7 (19)
Africa	3 (8)
Australia	1 (3)
Asia	1 (3)
***Income status***	
High income	33 (89)
Upper-middle income	3 (8)
Low-middle income	1 (3)
***Definition of LHS***	
IoM definition	9 (24)
Non-IoM definition with citation	11 (30)
No definition	17 (46)
***Study type***	
Empirical	20 (54)
Non-empirical	17 (46)
***Setting***^a^	
Primary care	32 (86)
General practice	14 (38)
Community health	13 (35)
Multidisciplinary	9 (24)
Allied health	5 (14)
Emergency department	7 (19)
***LHS components***	
Science and informatics	34 (92)
Continuous learning culture	31 (84)
Structure and governance	28 (76)
Patient-clinician partnerships	17 (46)
Incentives	17 (46)
***Focus on adopted or recommended LHS components***^b^	
Reports on adopted LHS components or models	32 (86)
Primary care	28 (76)
Emergency department	6 (16)
Recommends LHS components or models	5 (14)
Primary care	4 (11)
Emergency department	1 (3)
***Factors influencing implementation***	
Barriers to implementation	21 (57)
Primary care	18 (49)
Emergency department	3 (8)
Facilitators of implementation	22 (59)
Primary care	19 (51)
Emergency department	3 (8)

*N* Total number of articles included in scoping review, *n* number of articles included in the frequency analysis, *IoM* Institute of Medicine, *LHS* Learning health system

^a^Two articles were set in both primary care and emergency department; Nine articles were classified in more than one primary care setting category

^b^Two articles report on adopted LHS components in both PC and ED settings

Various definitions were cited in the included articles to define or describe components of an LHS. The IoM definition was the most consistently used, cited in nine articles (24%) [38–46]. Three articles (8%) used the Agency for Healthcare Research and Quality (AHRQ) definition of an LHS [47–49], and 8 articles (27%) referred to other reports of LHSs to define the concept [30, 50–53], including that of Foley and colleagues [31], and Menear and colleagues [54, 55]. Seventeen articles (46%) did not include a definition of an LHS [32, 34, 56–60].

### Learning health system components across settings

Tables 2 and Supplementary Material 2 describe the LHS components across PC and ED settings that were extracted from the included studies. Of the 37 included articles, 32 (86%) explored LHS components or models within PC settings, including general practice (*n* = 14; 38%), community health (*n* = 13; 35%), multidisciplinary care (*n* = 9; 24%), and allied health (*n* = 5; 14%). Multidisciplinary settings included care-at-home models [34, 61], integrated care organisations or clinics [30, 54], and multidisciplinary community health services [43, 51]. Seven articles (19%) explored LHS components or models within the ED setting, and two articles (5%) explored LHSs in both the PC and ED setting.

In investigations of PC, most articles described LHSs that had been *adopted*, at least at pilot stage (*n* = 28; 76%), and four of these articles reported on the adoption of all five LHS components [30, 38, 48, 62]. Four PC-based articles (11%) *recommended* initiatives or innovations to facilitate LHS progression, and one of these articles recommended an LHS model that contained all five components [37].

Barriers to adopting LHSs in PC were reported in 18 articles (49%). Overarching barriers that prevented LHS progression in PC included difficulties scheduling the participation of clinicians and other intervention participants [32, 45–48, 50, 57] and limited resources (e.g., funding, staffing) to support interventions [32, 35, 44, 47, 55]. Facilitators of adopting LHS in PC were reported in 19 articles (51%). General facilitators included stakeholder buy-in [32, 34, 45, 46, 57] and forward planning [43, 46–48, 51, 55, 57] for interventions. Barriers and facilitators which applied to the adoption of specific LHS components in PC are described in the sections below. Two articles utilised a framework (Consolidated Framework for Implementation Research; CFIR) to explore barriers and facilitators associated with LHS explorations [47, 48].

Of the articles investigating ED settings, most reported on LHS components that had been *adopted* (*n* = 6; 16%), and one article (3%) made *recommendations* for the development and adoption of LHS components into ED. There were no articles that reported on an LHS model in an ED setting that contained all parts of the five-component framework. Barriers and facilitators associated with implementing LHS components in ED were reported in 3 articles (8%), all of which were specific to the LHS interventions conducted.

#### Science and informatics

*Science and informatics* was reported on in 34 articles (92%). In PC, systems and processes that leverage electronic health records (EHRs) were a common focus, aimed at improving medication safety [51], improving concordance with best practice guidelines [38, 39], facilitating quality improvement [35, 63], identifying high-risk patients [30], and streamlining clinical work [38, 39]. Web-based tools were also leveraged to improve evidence-based knowledge transfer between stakeholders [35, 63], and to build knowledge repositories to inform learning and practice [64].

Common barriers to embedding *science and informatics* interventions into PC included the complexity of data infrastructure [61], data standardisation and management issues within EHRs [34, 61], professional resistance to implementation [51], and underdeveloped relationships with information technology teams [34]. Facilitators included leadership buy-in for resource allocation [34], trusting relationships between healthcare professionals involved in implementation [51], and making iterative changes to process [51].

In ED settings, there was also a strong focus on utilising EHRs to improve decision-making accuracy based on patient data [65], guideline adherence [56], and critical care during COVID-19 [33, 49]. Similar to PC settings, there were several barriers to EHR use including unreliable or missing data, and a lack of information on social determinants of health (e.g., homelessness), both of which were crucial for decision-making [65, 66]. Facilitators of embedding *science and informatics* tools into EDs included local implementation adapted to contextual factors, and the involvement of clinicians and community stakeholders in the continual development of interventions [49, 65].

#### Continuous learning culture

*Continuous learning culture* was reported in 31 articles (84%). Within PC, continuous learning most frequently centred on the creation of teams or learning collaboratives to facilitate communication, disseminate implementation results, share learnings [53] and build leadership to drive implementation [32, 35, 40]. Barriers to creating a *continuous learning culture* in PC included time constraints for clinicians [46], communication issues between healthcare professionals [37, 42], a lack of understanding of the respective roles and responsibilities of team members [50, 59, 62], and challenges with technology use [61]. Facilitators included regular meetings to ensure alignment and reduce the risk of misunderstanding [46], ‘huddles’ to share learnings [48], and mentorship to generalise findings between contexts [32] and to upskill leaders [61].

In ED settings, continuous learning and improvement focused primarily on leveraging real-time data collection platforms to improve clinical decision-making [36, 65]. One study reported a lack of insight into barriers to implementing these platforms, potentially due to the limited number of high-quality interventions in ED that examine implementation amidst time pressures and information overload [65]. Learning communities for knowledge sharing were also explored within the ED and were facilitated by strong relationships among clinicians and between professional organisations [51].

#### Structure and governance

*Structure and governance* was reported on in 28 articles (76%). Within PC, *structure and governance* primarily involved the creation of committees for oversight of intervention development and implementation [45, 55], many of which included patients and community members [48]. Structures to engage and involve local and national leadership (e.g., through partnerships) were both adopted and recommended in future models to facilitate quality improvement interventions [35, 37, 41, 50]. Protocols to guide the coordination and implementation of LHS interventions were an additional focus [48]. In ED settings, partnership structures were also leveraged to strengthen research and collaboration [33, 41, 63], including leadership to scale implementation [49], and planned protocols to guide intervention delivery [43].

Barriers to implementing *structure and governance* were mostly reported in PC settings, and included lengthy time periods waiting for ethics approval [46], difficulties securing funding [46], and varying barriers and policies across implementation contexts [37, 63]. Facilitators included formal leadership training and mentorship models [32, 61], formal strategic planning [57], and strong ongoing relationships with stakeholders [33].

#### Patient-clinician partnerships

*Patient-clinician partnerships* was examined in 17 articles (46%). In PC, *patient-clinician partnerships* mostly related to the inclusion of patients in governance structures, such as advisory committees, to identify patient and community priorities during intervention planning and implementation [43, 45]. Patient experiences and preferences were assessed primarily to inform LHS exploration or development [50, 53, 59, 62], rather than to measure patient satisfaction or preferences for care pathways. Barriers to involving patients in LHS interventions included issues recruiting and receiving feedback from patients [62] and lack of adequate communication processes between patients and implementation teams [48]. Facilitators included planning better communication strategies [48] and tailoring appointment lengths for greater patient contact [47, 48].

In the ED setting, only one article focused on patient engagement and empowerment in implementing a joint PC-ED based intervention [63]. Person-centredness was most frequently about ensuring patients and families received timely information and support [50, 59, 62], rather than involving patients and communities in LHS interventions. No articles reported on implementation factors associated with patient-clinician partnerships in ED.

#### Incentives

*Incentives* was assessed in 17 articles (46%). In PC, *incentives* were most commonly directed toward prioritising high value rather than high volume care [38, 52, 54], followed by data sharing and transparency [40, 50, 62]. Achieving stakeholder buy-in was also emphasised [32, 46] as well as the use of incentives to encourage training for healthcare professionals [42]. Barriers to successfully utilising incentives included inconsistently applied financial incentives among clinicians and other staff [38] and challenges to ascertaining stakeholder preferences in data availability [62]. Facilitators identified included ongoing discussions about appropriate incentive systems [38] and better planning with stakeholders around the availability of data from LHS interventions [62].

In ED, *incentives* was advocated in one article, for the purpose of supporting quality improvement in medication safety [49]. Quality incentives could be facilitated by clinician certification requirements, leadership endorsement, and opportunities to join learning collaboratives [49].

### Quality assessment

Eighteen studies were appraised using the MMAT. Of these, 10 (56%) were qualitative, four (22%) were quantitative descriptive studies, three (17%) were mixed methods, one was a quantitative randomized controlled trial, and one was a quantitative non-randomized study. One study was appraised using the JBI critical appraisal checklist for text and opinion papers. Two studies were appraised using the SANRA tool. All included studies were deemed to be of high-quality following appraisal. Thirteen studies were unable to be appraised with existing tools but were included in the review as they were deemed by the authorship team to show low risk of bias. Details of quality appraisal can be found in Supplementary Material 4.

## Discussion

This review explored how, and the extent to which, LHS principles have been applied to the front lines of care in support of health system preparedness, and the barriers and facilitators to doing so. Although there has been considerable progress with adoption of LHSs within the last five years, this research has been largely focused on PC settings. PC encompasses a broad array of services and settings, and multidisciplinary care has been highlighted as crucial for improved patient outcomes amidst growing disease burden and ageing populations [67, 68]. Indeed, many articles included in the current review reported multidisciplinary interventions [30] and other alternative care models to standard general practice [54], which appear to be increasingly popular. LHSs in EDs are beginning to emerge, but the focus on acute and urgent care in these settings, compounded by time and space constraints, can challenge attempts to improve health system efficiency and quality [69].

*Science and informatics* was the most frequently assessed LHS component across PC and ED settings, echoing previous work on LHS schematic frameworks [9]. Cycles of data collection, analysis, and feedback and the use of evidence-based methods were consistent themes across the included articles, with the ultimate aims of improving system performance and/or care outcomes [51, 63]. This improved system performance will have the added effect of reducing the carbon footprint of the PC and ED settings, an important component of future proofing the front lines of care [70, 71]. The complexities of data management tended to require extensive resourcing across PC and ED settings [41, 65], and were frequently enabled by strong relationships [50], regular communication [49], and governance teams [54]. These factors should be considered when planning to prepare PC and ED for future challenges.

Evidently, exploration of the ‘human’ aspects of LHSs [9] has increased overall in the last five years, through greater focus on learning communities, human interactions with technology, and patient involvement. *Incentives* and *patient-clinician partnerships,* however, were assessed almost 50 percent less frequently than the other three LHS components, despite being crucial for achieving the ‘buy-in’ needed to make technical LHS processes possible in both PC [32] and EDs [49]. Incentives create necessary willingness and motivation for individuals (e.g., healthcare professionals, managers, patients) to participate in interventions, which is important for ensuring the integrity of data obtained [46]. Furthermore, patients are increasingly viewed as key actors in healthcare, and LHS efforts should utilise the numerous frameworks for patient involvement that have been developed [72]. This is particularly relevant in the context of preparing the front lines of care for future pandemics and climate change, both of which will directly impact patients globally. These patients have a right to be involved in future-proofing the system which will ultimately protect their wellbeing in a changing world.

Although many barriers and facilitators to LHS progress were reported in the articles reviewed, few utilised implementation frameworks to systematically understand the factors influencing LHS adoption [47, 48]. Implementation science models and frameworks aim to improve the uptake of evidence into practice, and thus align closely with the goals of LHSs [7, 73]. For example, implementation science has been successfully utilised in PC to identify factors affecting LHS adoption, and inform implementation strategies tailored to local settings [48]. In ED settings, implementation science has only recently begun to gain traction [74], but has thus far not been utilised for LHS approaches. Implementation science tools should be exploited when planning LHS approaches to better understand how incentives, partnerships, governance, informatics, and learning can be embedded into healthcare organisations and workplaces [7, 73].

### Strengths and limitations

This review was the first to identify the breadth and range of LHS approaches that have been developed and adopted to improve care at the front lines. The findings highlighted various methods and conceptualisations of LHS components in PC and ED, and pointed to areas in which further research is warranted.

Although our search strategies were designed to broadly encompass LHS approaches, it is possible that the articles reviewed most frequently reported on effective interventions rather than those considered less effective, but no less useful, approaches to LHS development and implementation. Furthermore, most articles reviewed were published in high-income countries. It is possible that searches unlimited by language, and designed to capture challenges relating to LHS development, might have revealed more articles reporting from lower-income countries.

## Conclusions

The creation of LHSs at the front lines of care is essential for future-proofing against the challenges and risks facing health systems. While there has been greater attention placed on the ‘human aspects’ of LHSs, s*cience and informatics* remains the most frequently assessed component, and *incentives* and *patient-clinician partnerships* have been least examined. Implementation science should be utilised to better understand the factors influencing LHS adoption, so that increasingly stronger and more adaptable LHSs can be created – LHSs that are prepared for the pressures of future pandemics and climate change.

### Supplementary Information

Supplementary Material 1.

Supplementary Material 2.

Supplementary Material 3.

Supplementary Material 4.

## Data Availability

Data supporting these research findings are available in supplementary material and further inquiries can be directed to the corresponding author.

## Abbreviations

IoM: Institute of Medicine
LHS: Learning health system
PC: Primary care
ED: Emergency department
EHR: Electronic health record
