# Supplementary material for: Learning health systems on the front lines to strengthen care against future pandemics and climate change: a rapid review
Source: BMC Health Serv Res. 2024 Jul 22;24:829. doi: 10.1186/s12913-024-11295-3 (PMC11265124; doi:10.1186/s12913-024-11295-3)
Supplement: Supplementary file 1 — Supplementary Material 1. [file 12913_2024_11295_MOESM1_ESM.docx]

**Supplementary Material 2. Embase search strings**

*Embase search string for learning health systems in primary care*

((“Primary Medical Care/” OR “General Practice/” OR “primary care”) AND (“Learning Health System/” OR “learning health system*”))

Limits: English language; published from 1st January 2018 to 14th March 2023

*Embase search string for learning health systems in emergency departments*

((“Emergency Ward/” OR “emergency department*” OR “emergency room*”) AND (“Learning Health System/” OR “learning health system*”))

Limits: English language; published from 1st January 2018 to 14th March 2023
