# Supplementary material for: Learning health systems on the front lines to strengthen care against future pandemics and climate change: a rapid review
Source: BMC Health Serv Res. 2024 Jul 22;24:829. doi: 10.1186/s12913-024-11295-3 (PMC11265124; doi:10.1186/s12913-024-11295-3)
Supplement: Supplementary file 2 — Supplementary Material 2. [file 12913_2024_11295_MOESM2_ESM.docx]

***Supplementary table 1***

| **Authors** | **Country** | **Article type** | **Adopted or recommended** | **Setting** | **Implementation factors reported** | **Learning health system components** |
| --- | --- | --- | --- | --- | --- | --- |
| Abraham (2021) | USA | Empirical | Adopted | PC (multi-disciplinary) | Barriers and facilitators | - *Science & informatics:* Complexity of clinical data infrastructure contributes to problems locating and managing data for decision-making. - *Continuous learning culture:* Effective leadership is hindered by siloed priorities and practices, pace of change, complexity of data management, overreliance on technology for communication, and gaps in leadership training; effective leadership is facilitated by soft skills development (e.g., listening skills), face-to-face interaction, and mentorship. - *Structure & governance:* Formal leadership training and mentorship were recommended as facilitators of leading within the medical home model. |
| Archambault (2020) | Canada | Non-empirical | Adopted | ED & PC (community health) | N/A | - *Science & informatics:* Utilised a “Wiki-suite” (free web-based database containing evidence-based knowledge tools) to engage multiple stakeholders. - *Patient-clinician partnerships:* A subcommittee of caregiver and patient representatives from participating sites engaged and feedback solicited from a patient representative. - *Continuous learning culture:* Identified champions to lead local implementation; regular meetings with local hospital and community-based clinicians and decision-makers; online training for knowledge tool use, including personalised support; encouraged knowledge sharing across centres - *Structure & governance:* Executive committee to oversee study, including community-based clinicians and ED clinicians, and review implementation progress; subcommittee of caregiver and patient representatives from participating sites. |
| Awoonor-Williams (2022) | Ghana | Non-empirical | Adopted | PC (community health) | Barriers and facilitators | - *Science & informatics:* Digital and social medial used to share best practices, identify champions of change, and facilitate learning across governance levels. - *Continuous learning culture:* Build leadership at community levels to empower stakeholders and mobilise support; integrate project communication into routine reports, policy pronouncements and guidelines. - *Structure & governance:* Each stage was governed by steering committees chaired by the Ghana Health Service Director General. |
| Baynes (2022) | USA | Non-empirical | Adopted | PC (community health) | N/A | - *Science & informatics:* Advocates for embedded implementation research (EIR) as an evidence-based intervention to integrate scientific inquiry within practice; decision makers take a prominent role in research to bridge the evidence-policy-practice gap. - *Continuous learning culture:* Partnerships between health ministries, embedded scientists from primary care systems, and local level implementation teams establish an ongoing culture of knowledge sharing and learning. |
| Branch Elliman (2023) | USA | Non-empirical | Recommended | ED | Barriers and facilitators | - *Science & informatics*: Advocates for the creation of automated infection surveillance strategies, enabled by EHR and genomics technologies, to revolutionize the practice of infection detection, prevention and control. - *Continuous learning culture*: Data collected in near real time can be analysed and applied in clinical settings to improve bedside care and infection control policies. |
| Brannon (2018) | USA | Empirical | Adopted | PC (multi-disciplinary, community heath) | N/A | - *Science & informatics:* Predictive modelling with EHR data used to identify high-risk individuals to receive a care coordination intervention to reduce ED utilization. - *Patient-clinician partnerships:* One intervention component centred on communicating care plan to patient directly, as well as streamlining management plans across care providers. - *Incentives:* Trialled payment system changes that incentivised local partnerships for health and social care integration. - *Continuous learning culture:* Adopted learning cycles to leverage data for problem-solving, translate knowledge into practice through intervention, and assess data generated from the intervention to drive further iterative learning. - *Structure & governance:* Multidisciplinary stakeholders including representatives from the health systems, care coordinating organizations, and community members, met on a monthly basis to evaluate different aspects of the process. |
| Burdick (2022) | USA | Empirical | Adopted | PC (multi-disciplinary, community health) | Barriers and facilitators | - *Science & informatics:* Use of best practice guidelines for all activities, subject matter experts, real-time data capture, EHR clinical decision support alerts, data visualization techniques, and interoperable frameworks. - *Incentives:* Implemented primary care professional payments based on relative value unit payments with penalties for readmissions - *Continuous learning culture:* Disseminated findings at local meetings, held in-person and virtual Learning Collaboratives to facilitate sharing of best practices, and identified intervention champions. - *Structure & governance:* Development of the Improvement Support Team to, together with organisational leaders, develop team and project charters, and standardize and improve workflows and tools for staff; established stakeholder engagement mechanisms (e.g., committees) |
| Campbell (2021) | USA | Non-empirical | Adopted | PC (multi-disciplinary) | Barriers and facilitators | - *Science & informatics:* Evidence-based quality measure design and implementation into EHR for depression screening; involves defining quality measure criteria and validating them against actual outcomes of clinical practice - *Continuous learning culture:* Defining the EHR quality measure algorithm is an iterative process as criteria are validated, and recording of measure-related activity is improved; regular interdisciplinary team rounds held to review quality measures and discuss better achievement of target. - *Structure & governance:* Regularly scheduled interdisciplinary team rounds to manually review screening completion. |
| Cornick (2018) | South Africa | Non-empirical | Adopted | PC (general practice) | Barriers and facilitators | - *Science & informatics:* Developed a global guide of evidence-based diagnostic, screening and management recommendations adaptable across countries, aligned with WHO guidance. - *Incentives:* Identified the importance of stakeholder buy-in to ensure successful localisation and then adoption of intervention at the outset. - *Continuous learning culture:* Intervention championed by local clinicians with a strong vision for integrated primary care; some local changes absorbed into the global update and then implemented in other localisations. - *Structure & governance:* Provision of mentorship model of localisation to ensure that the programme is tailored to local resource constraints. |
| Dammery (2023) | Australia | Empirical | Adopted | PC (general practice) | Barriers and facilitators | - *Science & informatics:* Digital platforms used to aid in everyday clinical processes included EHRs, digital health support tools, and patient-directed smartphone app. - *Patient-clinician partnerships:* Methods for current and future patient involvement within the practice included focus groups for regular patient feedback, online review platforms, and a biannual quality improvement survey. - *Incentives:* Financial incentives (i.e., paying a salary to doctors) reduces low value care. - *Continuous learning culture:* Leadership and management valued learning for all, reflected in weekly update emails on best practice guidelines, events, and achievements; university environment presented opportunities to engage in teaching, supervision, and learning. - *Structure & governance:* Multidisciplinary working groups for chronic disease management involving clinical and non-clinical staff, and a mentoring system between doctors, nurses, and administrative staff. |
| Delvaux (2018) | Belgium | Non-empirical | Recommended | PC (general practice) | N/A | - *Science & informatics:* Electronic data capture system developed which enables automatic primary care data extraction to reduce clinician administrative burden and centralises clinical registry data; linkage of health data and collection of patient-specific data from EHRs to facilitate clinical and quality improvement research. |
| Deruiter (2022) | Canada | Non-empirical | Adopted | PC (general practice, community health) | N/A | - *Science & informati*cs: Use of a data collection platform to generate new knowledge (e.g., of barriers to access) that can eventually be implemented into practice in a knowledge-to-action cycle. - *Patient-clinician partnerships:* Regular patient assessment, behavioural counselling and personalised treatments provided. - *Continuous learning culture:* Continuous capacity building via fortnightly conferences that provide education and support program implementation; educational online modules to provide strategies for working with specific populations. - *Structure & governance:* Provision of access to NRT medications at no cost to the patient, thereby reducing inequities associated with access to pharmacological aids |
| Golden (2019) | USA | Empirical | Adopted | PC (allied health) | Barriers and facilitators | - *Patient-clinician partnerships:* Patient feedback on services preferences obtained through survey which was used to inform local leadership. - *Incentives:* Facilities were sent their data which were benchmarked against national aggregated data; national findings and data were presented to sites in order to invite discussion and communication. - *Structure & governance:* policy issues of interest were proposed at a national level in project development phase and used to refine survey questions; local leaders engaged for support with letters provided in implementation toolkit; study findings used to create opportunities to liaise with leadership staff, inform clinic policy/practices and/or apply for funding. |
| Groenhof (2020) | The Netherlands | Empirical | Adopted | PC (general practice) | N/A | - *Science & informatics:* Linkage of health data to identify patients at high risk of cardiovascular disease, communicate with specialists, and thus improve the cardiovascular care continuum. |
| Hek (2022) | The Netherlands | Empirical | Adopted | PC (general practice) | Barriers and facilitators | - *Science & informatics:* Development of an innovative research infrastructure (Benefit, Risk, and Impact of Medication Monitor (BRIMM)) which combines EHR data and patient-reported outcome data used to explore specific diseases or treatments, comorbidities medication interactions, patient perspectives, and economic evaluations. - *Patient-clinician partnerships:* Trial and implementation of a patient panel for input and feedback, patients provided with access to their questionnaires to enable discussion with their healthcare provider; Incorporation of patient feedback loop into future models recommended. - *Incentives:* Transparency fostered through having linked data resulting from this registry available for use by parties; GPs received feedback on participating patients in their practice in comparison with other practices. - *Continuous learning culture:* Developed research infrastructure that recommended feedback loops for GPs which included information on adverse effects, healthcare utilisation and adherence to treatment. - *Structure & governance:* Use of platform can be extended to provide information for policymakers and the health system. |
| Hunt (2021) | USA | Non-empirical | Adopted | ED | Barriers and facilitators | - *Science & informatics:* Virtual education resource operated via telehealth recorded learning sessions to serve as a public repository; real-time interaction through chat and Q&A functions. - *Continuous learning culture:* Peer-to-peer learning networks that allow real time sharing of information, scaled up across the US. - *Structure & governance:* Platform for collaboration with professional societies and organisations; findings from participant polling used to inform decision-making and policy at organisational level and federal agencies. |
| Jeffries (2018) | UK | Empirical | Adopted | PC (general practice, allied health) | Barriers and facilitators | - *Science & informatics:* Pharmacist-led intervention designed to identify and address potentially hazardous prescribing using a real-time surveillance dashboard identifying prescribing and monitoring safety indicators, EHR review, and risk resolution with general practice staff. - *Patient-clinician partnerships:* Patients could be present and involved during sessions if desired. - *Continuous learning culture:* Regular meetings of pharmacists to share learning and develop novel approaches to working with the implemented dashboard in their practices. |
| Jones (2018) | USA | Empirical | Adopted | ED | N/A | - *Science & informatics:* Implementation of a digital tool that integrates individual patient data and risk assessments with guideline-based recommendations to assist workflow at a critical point in the clinical decision-making process; utilises electronic alerts with continuously updated patient data when the estimated likelihood of disease exceeds a certain threshold. - *Continuous learning culture:* Tool assists with decision making by screening all ED patients with chest imaging for evidence of pneumonia, alerting providers through continuously updated electronic ED patient tracking board. |
| Khanna (2020) | USA | Non-empirical | Adopted | PC (general practice) | N/A | - *Science & informatics:* Population health dashboard created to collate COVID-19 patient tests results and outcomes, and identify emerging trends to track patients, share data in real-time, and strategically plan resources (e.g., personal protective equipment). - *Patient-clinician partnerships:* Physicians initiated outreach to patient panels to meet individual patient needs; support provided to COVID positive patients through telehealth visits, including symptom management, medication refills and ensuring food and transport arrangements; improved access to COVID-19 clinics for patients through increased opening hours. - *Incentives:* Transparency fostered by disseminating local site data to leaders and frontline clinicians allowing benchmarking against national aggregated results. - *Continuous learning culture:* A leadership team was established by the Chairman to allow ideas to incubate, percolate, and translate into action items; physician and staff training with elbow supports was provided. |
| Li (2020) | China | Non-empirical | Recommended | PC (general practice) | Barriers and facilitators | - *Science & informatics:* Recommends integrated, centralised, and standardised clinical information technology systems that can securely capture, and selectively grant access to, data, and that enable informative insights on service quality and effectiveness. - *Patient-clinician partnerships:* Clinician training should include a patient-centred perspective with integration of patient goals and education on empathy and shared decision making. - *Incentives:* To motivate GPs to attend education training programs (e.g., certification), establish performance accountability to incentivise high-quality and high-value care; data on care quality should be made publicly available to increase accountability, engender trust, and drive improvement. - *Continuous learning culture:* Use digital learning platforms in training for the PC workforce; clinical practice guidelines need to be tailored for PC settings and contain feasible and affordable recommendations to facilitate improvement. - *Structure & governance:* National bodies could strengthen capacity of departments to monitor and provide feedback and support and ensure payments reward good performance; consideration of budget reform from fee-for-service to capitation payment method; federal government could set targets for the percentage of medical graduates who pursue postgraduate training in general practice. |
| McCreary (2022) | USA | Empirical | Adopted | ED & PC (allied health) | N/A | *Primary Care*   - *Science & informatics:* Rapid generation of practice-based evidence within the context of a quality improvement initiative evaluating EHR-embedded routine care. - *Patient-clinician partnerships:* Prescribers provided patients with relevant fact sheets and reviewed the information and policies with each patient and/or patient guardian/representative. - *Structure & governance:* Policy-driven structure; partnership with federal government to launch platform and optimise scale-up including utilising a multifaceted outreach campaign; partnership with government public health bodies, community leaders, and neighbouring health systems to increase awareness and referrals.   *Emergency Department*   - *Structure & governance:* Partnership with federal government to launch platform and optimise scale-up including utilising a multifaceted outreach campaign and expanding infusion capacity to multiple EDs. |
| McGuire (2019) | USA | Non-empirical | Recommended | PC (general practice) | N/A | - *Science & informatics:* Promote use of informatics and data through effective EHR use; employ clinical experts to teach EHR functionality; educate clinicians on digitation of diagnosis. - *Patient-clinician partnerships:* Team training should include the use of education resources to develop or support implementation of a patient experience curriculum with the goal to support patient relationships and experience. - *Incentives:* Provided financial incentives to encourage providers to complete EHR upgrade training, with additional incentives for structured coaching of colleagues and staff. - *Continuous learning culture:* Team-based learning with coaching that reinforces knowledge transfer and continuous learning; regular training updates to ensure EHR enhancement is communicated. |
| Myers (2018) | USA | Non-empirical | Adopted | PC (multi-disciplinary, community health) | Facilitators | - *Science & informatics:* Identifying and adapting evidence‐based methods to increase colorectal and lung cancer screening in primary care; review health system data on lung cancer screening and disparities. - *Patient-clinician partnerships:* Patients were involved through an advisory committee to review and adapt evidence-based interventions for cancer screening to fit the needs of populations. - *Continuous learning culture:* Continuous communication and learning was facilitated through emails, meetings and a web platform, allowing real-time sharing of knowledge. - *Structure & governance:* Program tailored to local resource constraints; coordinating team, a steering committee, and patient and stakeholder advisory committees. |
| Myers (2020) | USA | Non-empirical | Adopted | ED | N/A | - *Science & informatics:* All resuscitations recorded with a multicamera video system; communication enabled by video chat; resuscitation events documented by nurses in real time; clinical care notes updated in EHR. - *Patient-clinician partnerships:* Assistance provided to both the patient and family members either remotely via video chat or face-to-face; provide support to children based on age-associated coping. - *Continuous learning culture:* Quality improvement program encouraging ongoing learning through seamless digital communication, publicly accessible written guides on technology use, regular rounding to support process and system changes, and intermittent process reviews. - *Structure & governance;* collection of prospective data from the resuscitation physician leader immediately after each event; fortnightly review of videos, regular evaluation and assessment to identify areas of weakness; various roles transitioned to work remotely. |
| Nash (2022a) | Canada | Empirical | Adopted | PC (community health) | Barriers and facilitators | - *Science & informatics:* Identified that key to LHS development is good data quality on which to base decisions, requiring more proficient EHR functionality to capture desired data, greater standardisation of inputs into EHR, and real-time data to allow rapid decisions. - *Incentives:* Desire to improve care and engage in quality improvement best driven by stimulating intrinsic motivation and using incentives such as competition or peer pressure. - *Continuous learning culture:* Identified that quality improvement must be embedded into organisational culture and workflows, with clinical champions driving change, and adequate support to enable the LHS to be fully realised. - *Structure & governance:* Identified the need to have someone in a dedicated data support role as an enabler to embedding ongoing quality improvement. |
| Nash (2022b) | Canada | Non-empirical | Adopted | PC (community health) | Barriers and facilitators | - *Science & informatics:* Developed an information management committee responsible for protecting data privacy, ensuring data quality and standards, and providing technical support, to facilitate evidence-based planning and decision making. - *Patient-clinician partnerships:* Continuous involvement of clients, their caregivers, and the broader community members to identify their priorities and ensure meaningful research and quality improvement initiatives; use of patient-reported measures on experiences and outcomes. - *Continuous learning culture:* Ongoing evaluation plan to better understand the successes and challenges of the redesign; learning collaboratives launched to help a group of peers to work together to solve common problems and engage with quality improvement. - *Structure & governance:* Use of a robust committee structure that ensures productivity and prioritization of learning activities: Information management committee, steering committee, and executive-leader-led committee; reallocation of resources needed to operationalise the learning health system, including funding from academic researchers' grants for specific projects. |
| Nelson (2021) | USA | Non-empirical | Adopted | PC (multi-disciplinary) | Barriers and facilitators | - *Science & informatics:* Primary care analytics data platform (e.g. Medicare data, VHA data, census data) that allows for rapid analysis; primary care-related literature repository for rapid and efficient collection of targeted information. - *Incentives:* Funding policies that incentivise healthcare facilities to enrol veterans into primary care services. - *Continuous learning culture:* Monthly calls with the national network to share strategic goals, project information, results and dissemination; interactive presentations, national cyber-seminars, and peer-reviewed publications to share learnings. - *Structure & Governance:* Address and overcome barriers by assuring stable funding, gaining senior leadership support and developing clinical and research partner engagement; frequent and structured contact with leadership to promote mutual trust and bidirectional communication. |
| Neprash (2022) | USA | Empirical | Adopted | PC (general practice) | N/A | - *Science & informatics:* Intervention involved single sign-on access to a prescription drug monitoring program integrated into EHR allowing clinicians to review patients substance prescription and history; integration tool alerted clinicians to review when necessary, improving adherence to guidelines. - *Structure & governance:* Clinicians were legislatively mandated to query the integrated program before any initial prescription for opioids. |
| Palin (2020) | UK | Non-empirical | Adopted | PC (general practice) | Barriers | - *Science & informatics:* Intervention to optimise antibiotic prescribing in primary care enabled by a system capable of extracting and processing patient data and producing actionable results to practices. - *Incentives:* Points systems and payment schemes exist to incentivise the provision of quality care, where practices are financially rewarded based on how they are performing. - *Structure & governance:* Platform gives control to practices, equipping them to monitor and be accountable for their antibiotic prescribing; practices can defend their prescribing decisions to clinical commissioning groups that review practice performance. |
| Pestka (2022) | USA | Empirical | Adopted | PC (allied health) | Barriers and facilitators | - *Incentives:* As part of the primary care transformation, comprehensive medication management (CMM) was included as a covered service for all patients, facilitating its uptake. - *Continuous learning culture:* Barriers to integrating CMM into primary care practice included lack of clinic leadership, lack of team-based care, limited training for pharmacists, and resourcing constraints; facilitators included identifying CMM champions, and changing mindsets to prioritise the value of CMM. - *Structure & governance:* Importance of local leadership identified to address barriers at the clinical level and facilitate CMM uptake. |
| Pestka (2021) | USA | Empirical | Adopted | PC (allied health, multi-disciplinary) | Barriers and facilitators | - *Science & informatics:* Primary care transformation involved patients placed into a service bundle using an algorithm of EHR data which providers were able to override and reassign where appropriate. - *Patient-clinician partnerships:* Patient feedback sought which provided an understanding of the importance of communicating with patients, adjusting patient expectations and ensuring an understanding of patient needs at the start of the project. - *Incentives:* Tested value-based payment model where providers were compensated based on previous salaries, rather than productivity via relative value units in the traditional fee-for-service payment structure. - *Continuous learning culture:* Greater involvement of other care team members was instigated through assigning workgroups to focus on increasing the use of interprofessional services; changes and updates communicated through emails, staff meetings and a weekly newsletter; importance of communication between stations identified and emphasised. - *Structure & governance:* Shifting clinical roles and training staff so that care is delivered differently, for example having clinical pharmacists prescribing medication instead of primary care providers. |
| Porat (2019) | UK | Empirical | Adopted | PC (general practice, community health) | N/A | - *Science & informatics:* Aimed to collaboratively design, with key stakeholders, and evaluate a long-term stroke care intervention that could be integrated as part of the EHR system; utilised linked datasets including stroke registry data and general practice data. - *Patient-clinician partnerships:* Patients provided with a copy of their management plan, information on their predicted risk and selected treatments, and date of follow-up to motivate patient adherence; patient feedback sought on extent to which they believed management plan would be effective. - *Continuous learning culture:* The importance of up-to-date information was emphasised as a means to ensure suggested treatments aligned with the most up-to-date evidence. |
| Safaeinili (2019) | USA | Empirical | Recommended | PC (multi-disciplinary, community health) | N/A | - *Patient-clinician partnerships:* Utilisation of input from numerous stakeholders, including patients and families, in the development of the patient-centred care model; key components include focus on health (rather than disease), flexibility in types of appointments, and provision of services beyond traditional primary care. - *Structure & governance:* The ability to describe and evaluate a learning health system that gave structure to the challenges and facilitators was achieved by understanding the different inner setting layers of the health care system hierarchy (e.g., by understanding the experiences of both staff and executives at all levels). |
| Thandi (2021) | Canada | Empirical | Adopted | PC (multi-disciplinary, community health) | Barriers and facilitators | - *Science & informatics:* Primary care surveillance network extracts EHR data from consenting clinicians, standardizes data into common ontologies and terminologies and makes the data available for quality improvement, disease surveillance and research; Tested supervised machine learning techniques within the EHR database to validate their use in customising a list of frail patients. - *Incentives:* Potential for high extrinsic incentives identified including increased stature, goal sharing, changing practice, publications and dissemination activities. - *Continuous learning culture:* Practice-based research and learning networks in primary care contribute to producing and utilising knowledge to drive quality improvement. - *Structure & governance*: Identified the need for structural support and recognising differences in data sharing across provinces. |
| Van Rensburg (2022) | South Africa | Empirical | Adopted | PC (community health) | Barriers and facilitators | - *Science & informatics:* Routine tuberculosis (TB) data elements and indicators were captured at the facility level and district level to identify possible health system bottlenecks in TB care; strong focus on data to knowledge and knowledge to practice component of LHS. - *Continuous learning culture:* Utilising theory of change, a strategic process of engagement between stakeholders (researchers, policy makers, front line workers) was created to review and modify planning as new data becomes available; collaborative relationships with stakeholders identified as facilitating sustainability of LHS development. - *Structure & governance:* Policy and guidelines reviewed to highlight best practices for TB screening, diagnosis, infection control, treatment and follow-up; a theory of change map was developed to guide planning; a collaborative coordinating structure was formalised with stakeholders (i.e., a learning community) and focused on newly identified challenges. |
| Vandenberg (2020) | USA | Empirical | Adopted | ED | Barriers and facilitators | - *Science & informatics:* Medication safety program built around evidence-based clinical guidelines on potentially inappropriate medications (PIM); common EHR used across sites; clinical decision support including electronic order sets guiding providers away from PIMs and toward alternative treatments. - *Incentives:* Buy-in from providers was sought by having leadership announce the program to providers, and an ED peer champion approached providers directly; proposed a community–academic partnership potentially through quality incentives, to widen the learning system, demonstrating ongoing improvement required for physician certification. - *Continuous learning culture*: A core component of the program was monthly provider feedback including individual performance and peer benchmarking; information across sites disseminated through a common website; sites that encountered challenges could look to established methods and processes found to be successful across other sites. - *Structure & governance*: LHS was composed of three independent implementation teams coordinated through subcontracts by the principal investigator’s institution; Leadership across sites met biweekly and once yearly in person to pool implementation knowledge. |
| Yigzaw (2022) | Norway | Empirical | Adopted | PC (general practice) | Facilitators | - *Science & informatics:* Evaluates a privacy-preserving audit and feedback system in clinical settings which extracts data daily from the local EHR system to aggregate performance indicators for GPs to be computed locally; clinical and informatics experts developed a questionnaire assessing GPs perceptions of feedback. - *Incentives:* Provided a platform for GPs to learn the extent to which their prescribing deviated from the mean and thus was itself an incentive for quality improvement. - *Continuous learning culture:* Audit and feedback system on prescribing practices gave GPs the ability to compare their performance indicators with those of their peers, but could be considered distractive by clinicians. - *Structure & governance:* A third party qualified and trusted to follow protocol specifications coordinated the data without learning private information, in accordance with a standard Norwegian security model. |

UK, United Kingdom; USA, United States of America; ED, emergency department; PC, primary care; LHS, learning health system, EHR, electronic health record; GP, general practitioner
