# Supplementary material for: Learning health systems on the front lines to strengthen care against future pandemics and climate change: a rapid review
Source: BMC Health Serv Res. 2024 Jul 22;24:829. doi: 10.1186/s12913-024-11295-3 (PMC11265124; doi:10.1186/s12913-024-11295-3)
Supplement: Supplementary file 3 — Supplementary Material 3. [file 12913_2024_11295_MOESM3_ESM.docx]

Supplementary Material 3. Learning health system components assessed in included studies

|  | **LEARNING HEALTH SYSTEM COMPONENTS** | | | | |
| --- | --- | --- | --- | --- | --- |
|  | Science and informatics | Patient-clinician partnerships | Incentives | Continuous learning culture | Structure and governance |
| **STUDY** |  | | | | |
| Abraham (2021) | ✓ |  |  | ✓ | ✓ |
| Archambault (2020) | ✓ | ✓ |  | ✓ | ✓ |
| Awoonor-Williams (2022) | ✓ |  |  | ✓ | ✓ |
| Baynes (2022) | ✓ |  |  | ✓ |  |
| Branch Elliman (2023) | ✓ |  |  | ✓ |  |
| Brannon (2018) | ✓ | ✓ | ✓ | ✓ | ✓ |
| Burdick (2022) | ✓ |  | ✓ | ✓ | ✓ |
| Campbell (2021) | ✓ |  |  | ✓ | ✓ |
| Cornick (2018) | ✓ |  | ✓ | ✓ | ✓ |
| Dammery (2023) | ✓ | ✓ | ✓ | ✓ | ✓ |
| Delvaux (2018) | ✓ |  |  |  |  |
| Deruiter (2022) | ✓ | ✓ |  | ✓ | ✓ |
| Golden (2019) |  | ✓ | ✓ |  | ✓ |
| Groenhof (2020) | ✓ |  |  |  |  |
| Hek (2022) | ✓ | ✓ | ✓ | ✓ | ✓ |
| Hunt (2021) | ✓ |  |  | ✓ | ✓ |
| Jeffries (2018) | ✓ | ✓ |  | ✓ |  |
| Jones (2018) | ✓ |  |  | ✓ |  |
| Khanna (2020) | ✓ | ✓ | ✓ | ✓ |  |
| Li (2020) | ✓ | ✓ | ✓ | ✓ | ✓ |
| McCreary (2022) | ✓ | ✓ |  |  | ✓ |
| McGuire (2019) | ✓ | ✓ | ✓ | ✓ |  |
| Myers (2018) | ✓ | ✓ |  | ✓ | ✓ |
| Myers (2020) | ✓ | ✓ |  | ✓ | ✓ |
| Nash (2022a) | ✓ |  | ✓ | ✓ | ✓ |
| Nash (2022b) | ✓ | ✓ |  | ✓ | ✓ |
| Nelson (2021) | ✓ |  | ✓ | ✓ | ✓ |
| Neprash (2022) | ✓ |  |  |  | ✓ |
| Palin (2020) | ✓ |  | ✓ |  | ✓ |
| Pestka (2022) |  |  |  | ✓ | ✓ |
| Pestka (2021) | ✓ | ✓ | ✓ | ✓ | ✓ |
| Porat (2019) | ✓ | ✓ |  | ✓ |  |
| Safaeinili (2019) |  | ✓ |  |  | ✓ |
| Thandi (2021) | ✓ |  | ✓ | ✓ | ✓ |
| Van Rensburg (2022) | ✓ |  |  | ✓ | ✓ |
| Vandenberg (2020) | ✓ |  | ✓ | ✓ | ✓ |
| Yigzaw (2022) | ✓ |  | ✓ | ✓ | ✓ |
