# Supplementary material for: Learning health systems on the front lines to strengthen care against future pandemics and climate change: a rapid review
Source: BMC Health Serv Res. 2024 Jul 22;24:829. doi: 10.1186/s12913-024-11295-3 (PMC11265124; doi:10.1186/s12913-024-11295-3)
Supplement: Supplementary file 4 — Supplementary Material 4. [file 12913_2024_11295_MOESM4_ESM.docx]

Supplementary Material 4. Quality Appraisal for included studies

| **Narrative reviews - SANRA** | | |
| --- | --- | --- |
| Study First Author | Li | Branch Elliman |
| Justification of the article's importance for the readership | 2 | 2 |
| Statement of concrete aims or formulation of questions | 2 | 1 |
| Description of the literature search | 2 | 0 |
| Referencing | 2 | 1 |
| Scientific reasoning | 1 | 2 |
| Appropriate presentation of data | 2 | 2 |
| Total score (out of 12) | 11 | 8 |

|  | **Mixed methods appraisal tool (MMAT)** | | | | | | | | | |
| --- | --- | --- | --- | --- | --- | --- | --- | --- | --- | --- |
| Study First Author |  | Golden | Groenhof | Hek | Jeffries | Jones | McCreary | Abraham | Brannon | Burdick |
|  | S1. Are there clear research questions? | Yes | No | Yes |  | Yes | Yes | Yes |  |  |
|  | S2. Do the collected data allow to address the research questions? | Yes | Yes | Yes |  | Yes | Yes | Yes |  |  |
| Qualitative | 1.1. Is the qualitative approach appropriate to answer the research question? |  |  |  | Yes |  |  | Yes |  |  |
|  | 1.2. Are the qualitative data collection methods adequate to address the research question? |  |  |  | Yes |  |  | Yes |  |  |
|  | 1.3. Are the findings adequately derived from the data? |  |  |  | Yes |  |  | Yes |  |  |
|  | 1.4. Is the interpretation of results sufficiently substantiated by data? |  |  |  | Yes |  |  | Yes |  |  |
|  | 1.5. Is there coherence between qualitative data sources, collection, analysis and interpretation? |  |  |  | Yes |  |  | Yes |  |  |
| Quantitative randomised controlled trials | 2.1. Is randomization appropriately performed? |  |  |  |  |  | No |  |  |  |
|  | 2.2. Are the groups comparable at baseline? |  |  |  |  |  | Yes |  |  |  |
|  | 2.3. Are there complete outcome data? |  |  |  |  |  | No |  |  |  |
|  | 2.4. Are outcome assessors blinded to the intervention provided? |  |  |  |  |  | Unclear |  |  |  |
|  | 2.5 Did the participants adhere to the assigned intervention? |  |  |  |  |  | Unclear |  |  |  |
| Quantitative non-randomised | 3.1. Are the participants representative of the target population? |  | Yes |  |  |  |  |  |  |  |
|  | 3.2. Are measurements appropriate regarding both the outcome and intervention (or exposure)? |  | Yes |  |  |  |  |  |  |  |
|  | 3.3. Are there complete outcome data? |  | Yes |  |  |  |  |  |  |  |
|  | 3.4. Are the confounders accounted for in the design and analysis? |  | Unclear |  |  |  |  |  |  |  |
|  | 3.5. During the study period, is the intervention administered (or exposure occurred) as intended? |  | Yes |  |  |  |  |  |  |  |
| Quantitative descriptive | 4.1. Is the sampling strategy relevant to address the research question? | Yes |  |  |  |  |  |  | Yes | Yes |
|  | 4.2. Is the sample representative of the target population? | Yes |  |  |  |  |  |  | Yes | Yes |
|  | 4.3. Are the measurements appropriate? | Yes |  |  |  |  |  |  | Yes | Yes |
|  | 4.4. Is the risk of nonresponse bias low? | No |  |  |  |  |  |  | Yes | Yes |
|  | 4.5. Is the statistical analysis appropriate to answer the research question? | Yes |  |  |  |  |  |  | Yes | Yes |
| Mixed methods | 5.1. Is there an adequate rationale for using a mixed methods design to address the research question? |  |  | Yes |  | Yes |  |  |  |  |
|  | 5.2. Are the different components of the study effectively integrated to answer the research question? |  |  | No |  | Yes |  |  |  |  |
|  | 5.3. Are the outputs of the integration of qualitative and quantitative components adequately interpreted? |  |  | Unclear |  | Yes |  |  |  |  |
|  | 5.4. Are divergences and inconsistencies between quantitative and qualitative results adequately addressed? |  |  | No |  | No |  |  |  |  |
|  | 5.5. Do the different components of the study adhere to the quality criteria of each tradition of the methods involved? |  |  | Unclear |  | No |  |  |  |  |

|  | **Mixed methods appraisal tool (MMAT)** | | | | | | | | | | | |
| --- | --- | --- | --- | --- | --- | --- | --- | --- | --- | --- | --- | --- |
| Study First Author |  | Dammery | Nash | Neprash | Pestka | Pestka | Porat | Safaeinili | Thandi | Van Rensburg | Vandenberg | Yigzaw |
|  | S1. Are there clear research questions? | Yes | Yes | yes | Yes | yes | Yes | yes | Yes | Yes | yes | yes |
|  | S2. Do the collected data allow to address the research questions? | Yes | Yes | yes | Yes | yes | Yes | yes | Yes | Yes | yes | yes |
|  | 1.1. Is the qualitative approach appropriate to answer the research question? | Yes | Yes |  | Yes | yes | yes | yes | Yes |  | yes |  |
|  | 1.2. Are the qualitative data collection methods adequate to address the research question? | Yes | Yes |  | Yes | yes | yes | yes | Yes |  | yes |  |
|  | 1.3. Are the findings adequately derived from the data? | Yes | Yes |  | Yes | yes | yes | yes | Yes |  | yes |  |
|  | 1.4. Is the interpretation of results sufficiently substantiated by data? | Yes | Yes |  | Yes | yes | yes | yes | Yes |  | yes |  |
|  | 1.5. Is there coherence between qualitative data sources, collection, analysis and interpretation? | Yes | Yes |  | Yes | yes | yes | yes | Yes |  | yes |  |
|  | 2.1. Is randomization appropriately performed? |  |  | Yes |  |  |  |  |  |  |  |  |
|  | 2.2. Are the groups comparable at baseline? |  |  |  |  |  |  |  |  |  |  |  |
|  | 2.3. Are there complete outcome data? |  |  |  |  |  |  |  |  |  |  |  |
|  | 2.4. Are outcome assessors blinded to the intervention provided? |  |  |  |  |  |  |  |  |  |  |  |
|  | 2.5 Did the participants adhere to the assigned intervention? |  |  |  |  |  |  |  |  |  |  |  |
|  | 3.1. Are the participants representative of the target population? |  |  |  |  |  |  |  |  |  |  |  |
|  | 3.2. Are measurements appropriate regarding both the outcome and intervention (or exposure)? |  |  |  |  |  |  |  |  |  |  |  |
|  | 3.3. Are there complete outcome data? |  |  |  |  |  |  |  |  |  |  |  |
|  | 3.4. Are the confounders accounted for in the design and analysis? |  |  |  |  |  |  |  |  |  |  |  |
|  | 3.5. During the study period, is the intervention administered (or exposure occurred) as intended? |  |  |  |  |  |  |  |  |  |  |  |
|  | 4.1. Is the sampling strategy relevant to address the research question? |  |  |  |  |  |  |  |  |  |  | yes |
|  | 4.2. Is the sample representative of the target population? |  |  |  |  |  |  |  |  |  |  | yes |
|  | 4.3. Are the measurements appropriate? |  |  |  |  |  |  |  |  |  |  | yes |
|  | 4.4. Is the risk of nonresponse bias low? |  |  |  |  |  |  |  |  |  |  | yes |
|  | 4.5. Is the statistical analysis appropriate to answer the research question? |  |  |  |  |  |  |  |  |  |  | yes |
|  | 5.1. Is there an adequate rationale for using a mixed methods design to address the research question? |  |  |  |  |  |  |  |  | yes |  |  |
|  | 5.2. Are the different components of the study effectively integrated to answer the research question? |  |  |  |  |  |  |  |  | yes |  |  |
|  | 5.3. Are the outputs of the integration of qualitative and quantitative components adequately interpreted? |  |  |  |  |  |  |  |  | yes |  |  |
|  | 5.4. Are divergences and inconsistencies between quantitative and qualitative results adequately addressed? |  |  |  |  |  |  |  |  | Can't tell |  |  |
|  | 5.5. Do the different components of the study adhere to the quality criteria of each tradition of the methods involved? |  |  |  |  |  |  |  |  | yes |  |  |

| **Quality Assessment for text and opinion papers (JBI)** | | |
| --- | --- | --- |
| Study First Author | Baynes |  |
| 1. Is the source of the opinion clearly identified? | Yes |  |
| 2. Does the source of opinion have standing in the field | Yes |  |
| 3. Are the interests of the relevant population the central focus of the opinion? | Yes |  |
| 4. Is the stated position the result of an analytical process, and is there logic in the opinion expressed? | Yes |  |
| 5. Is there reference to the extant literature? | Yes |  |
| 6. Is any incongruence with the literature/sources  logically defended? | N/A |  |
